# Supplementary figures and images for: Cytoplasmic circular dsDNA is a key constituent of stress granules
Source: eLife. 2026 Jul 13;15:RP111336. doi: 10.7554/eLife.111336 (PMC13363216; doi:10.7554/eLife.111336)

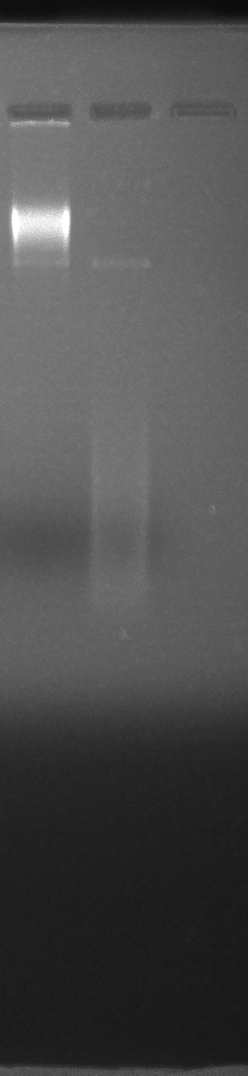

Supplement: Figure 1—source data 1. [file elife-111336-fig1-data1.zip › Figure 1-source data 1.tif]

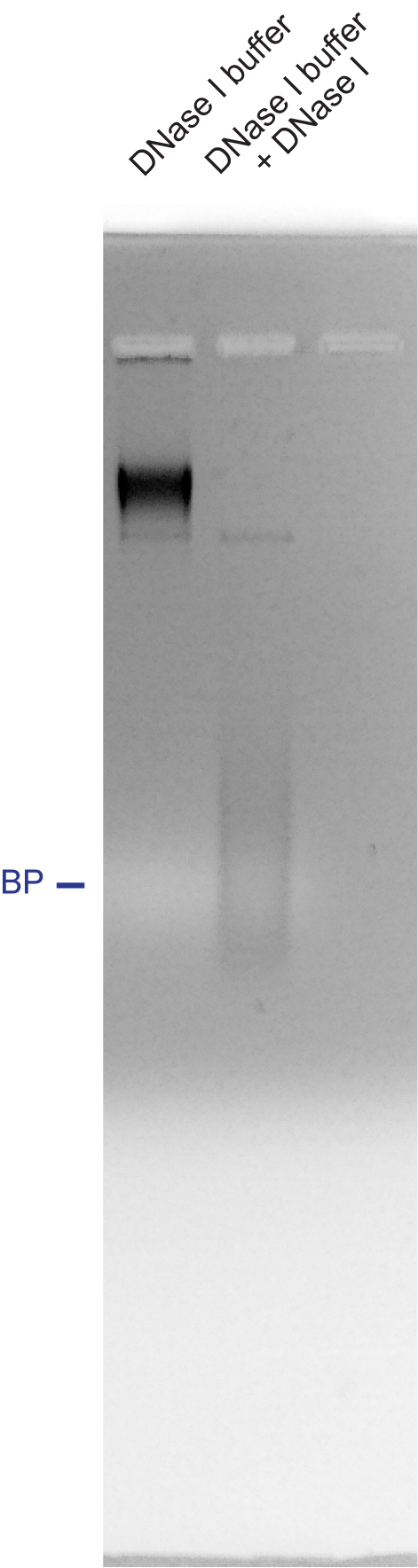

Supplement: Figure 1—source data 2. [file elife-111336-fig1-data2.zip › Figure 1-source data 1_labeled.tif]

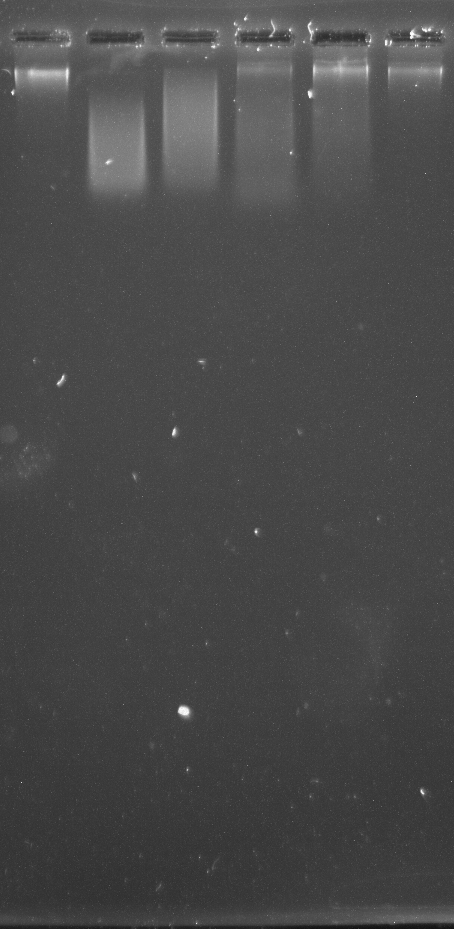

Supplement: Figure 1—source data 3. [file elife-111336-fig1-data3.zip › Figure 1-source data 2.TIF]

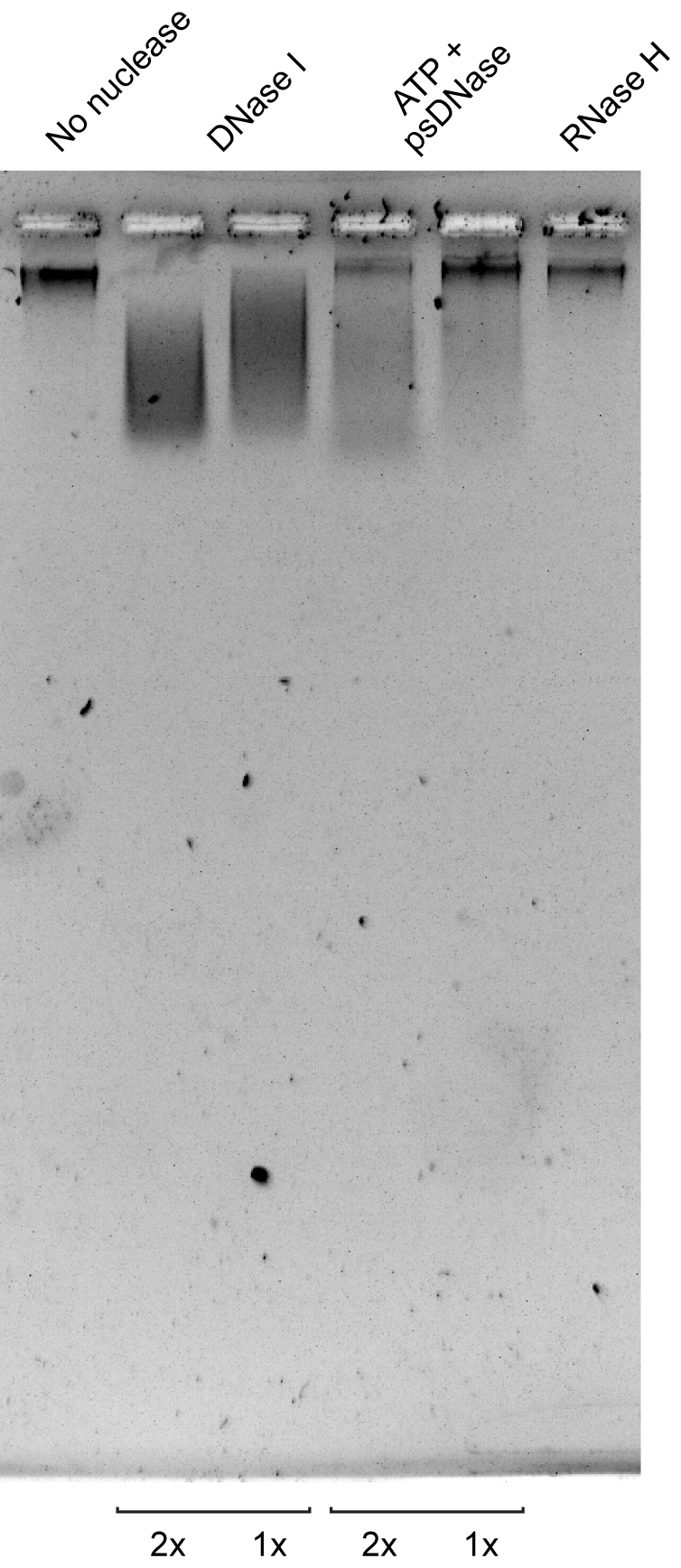

Supplement: Figure 1—source data 4. [file elife-111336-fig1-data4.zip › Figure 1-source data 2_labeled.tif]

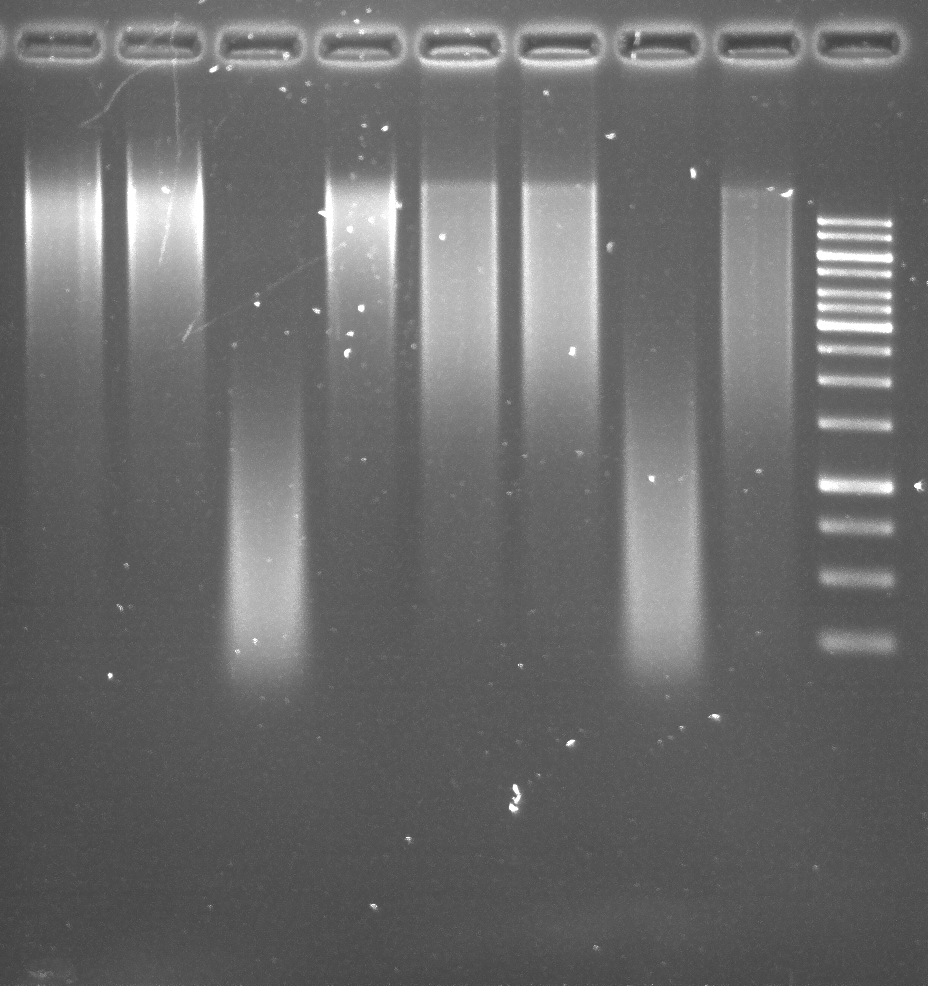

Supplement: Figure 1—source data 5. [file elife-111336-fig1-data5.zip › Figure 1-source data 3.TIF]

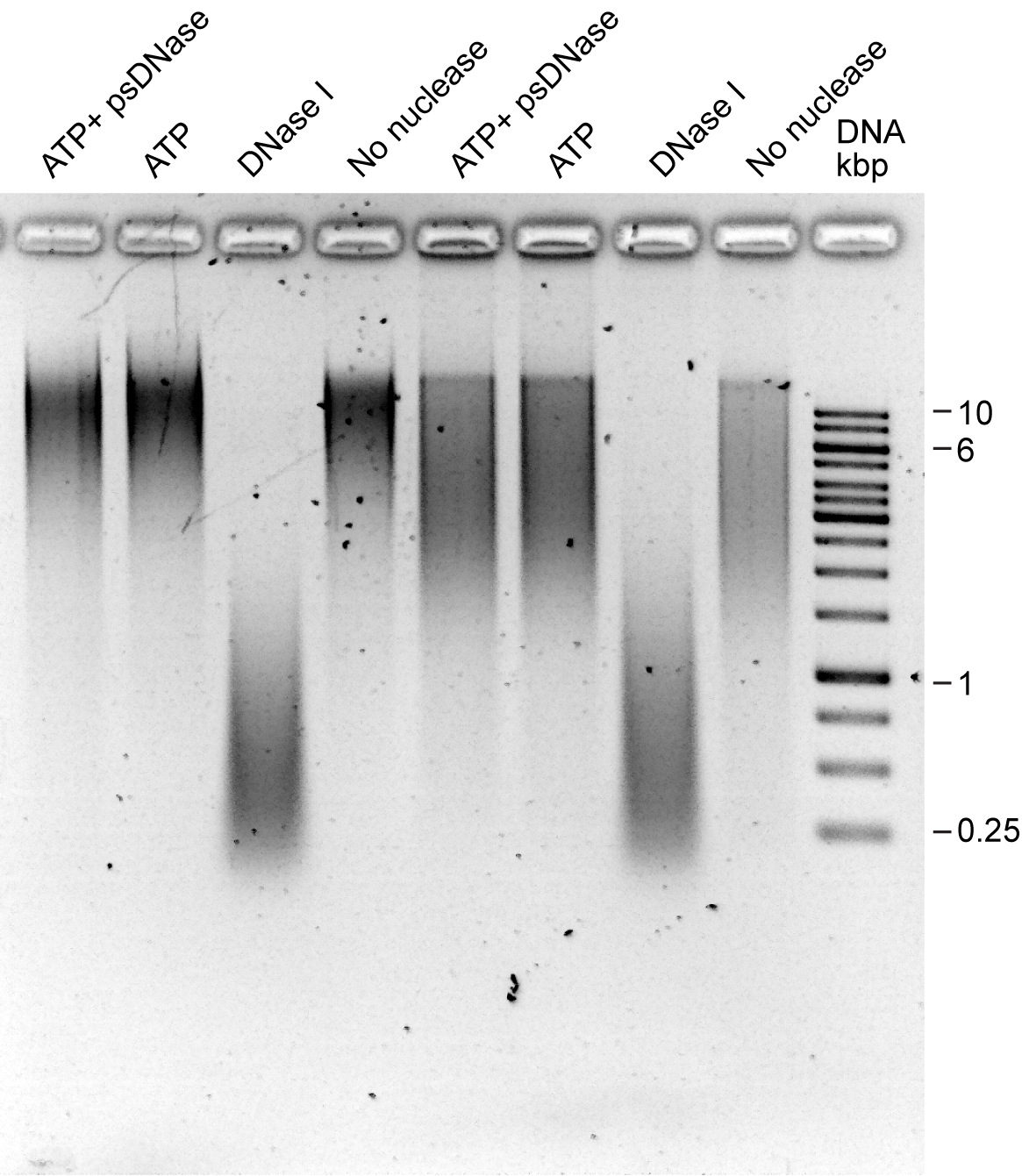

Supplement: Figure 1—source data 6. [file elife-111336-fig1-data6.zip › Figure 1-source data 3_labeled.tif]

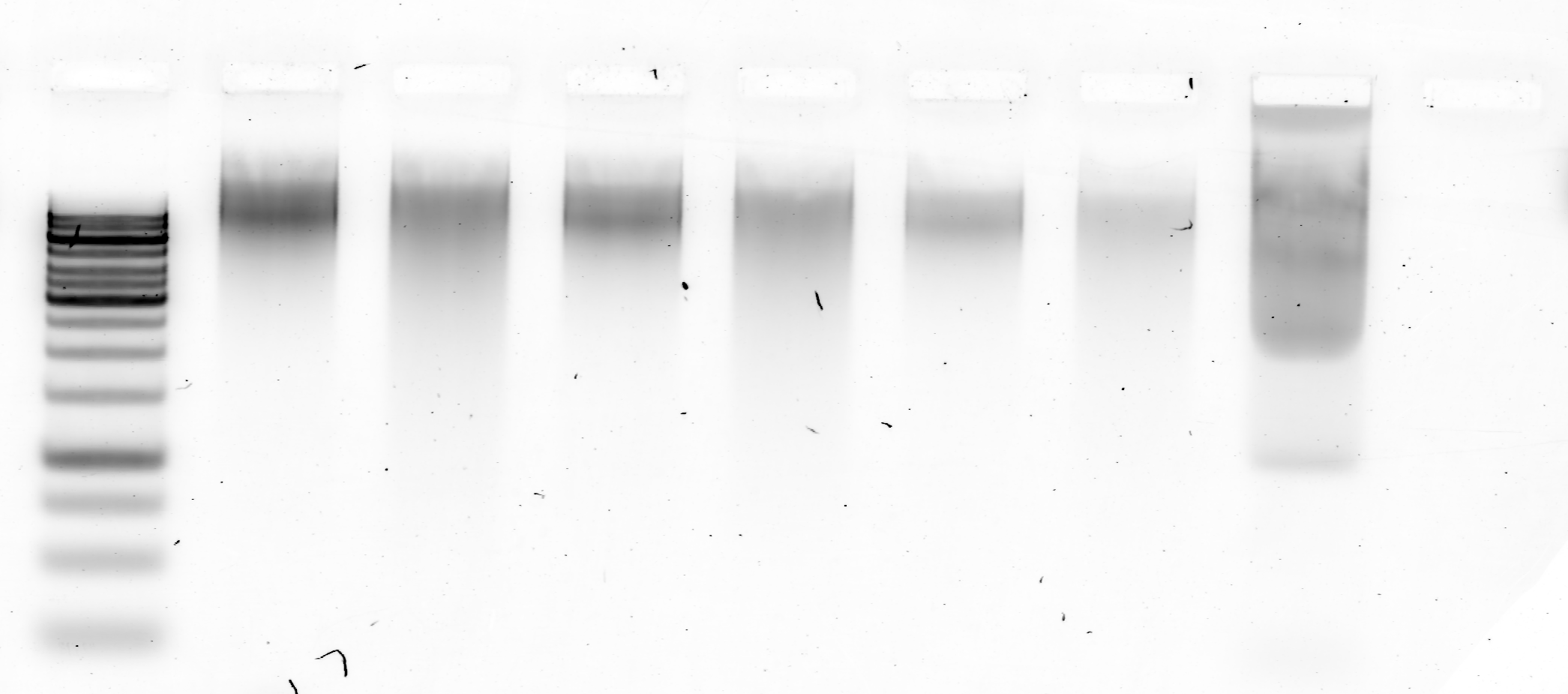

Supplement: Figure 1—source data 7. [file elife-111336-fig1-data7.zip › Figure 1-source data 4.tif]

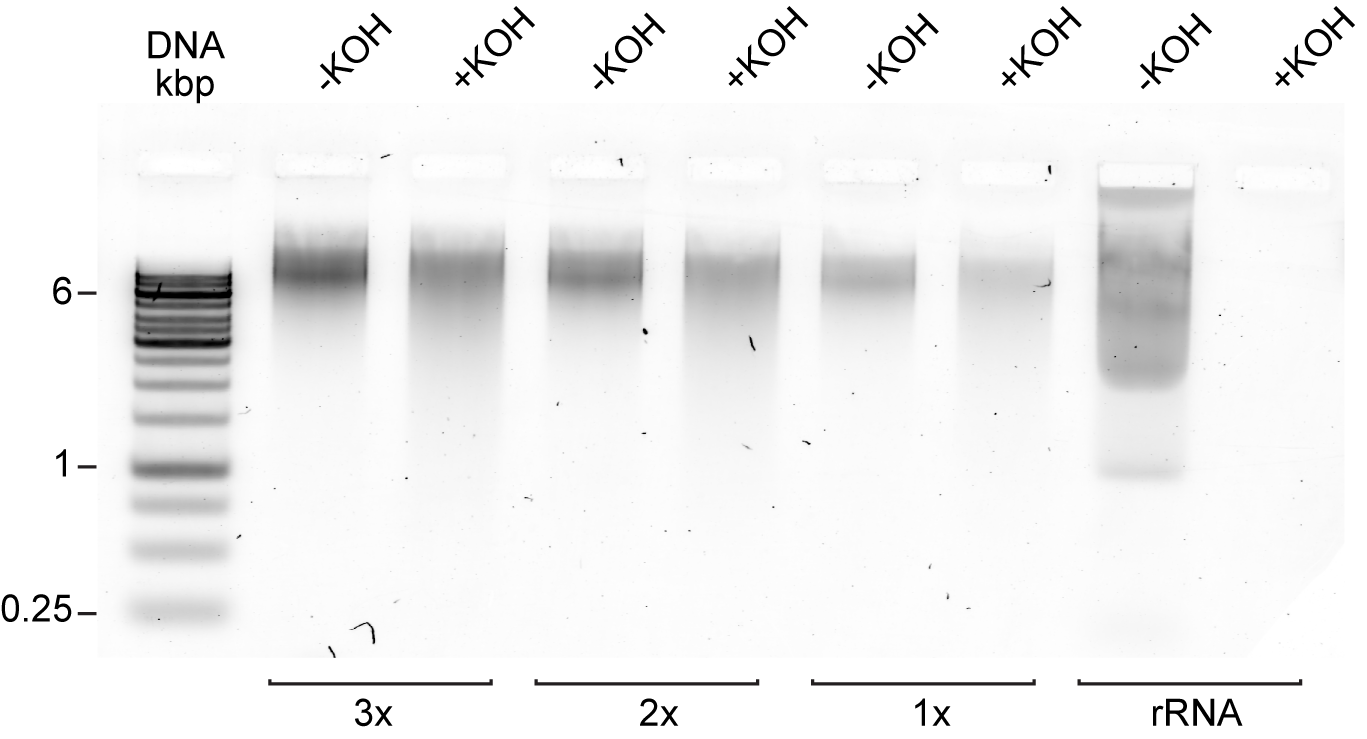

Supplement: Figure 1—source data 8. [file elife-111336-fig1-data8.zip › Figure 1-source data 4_labeled.tif]

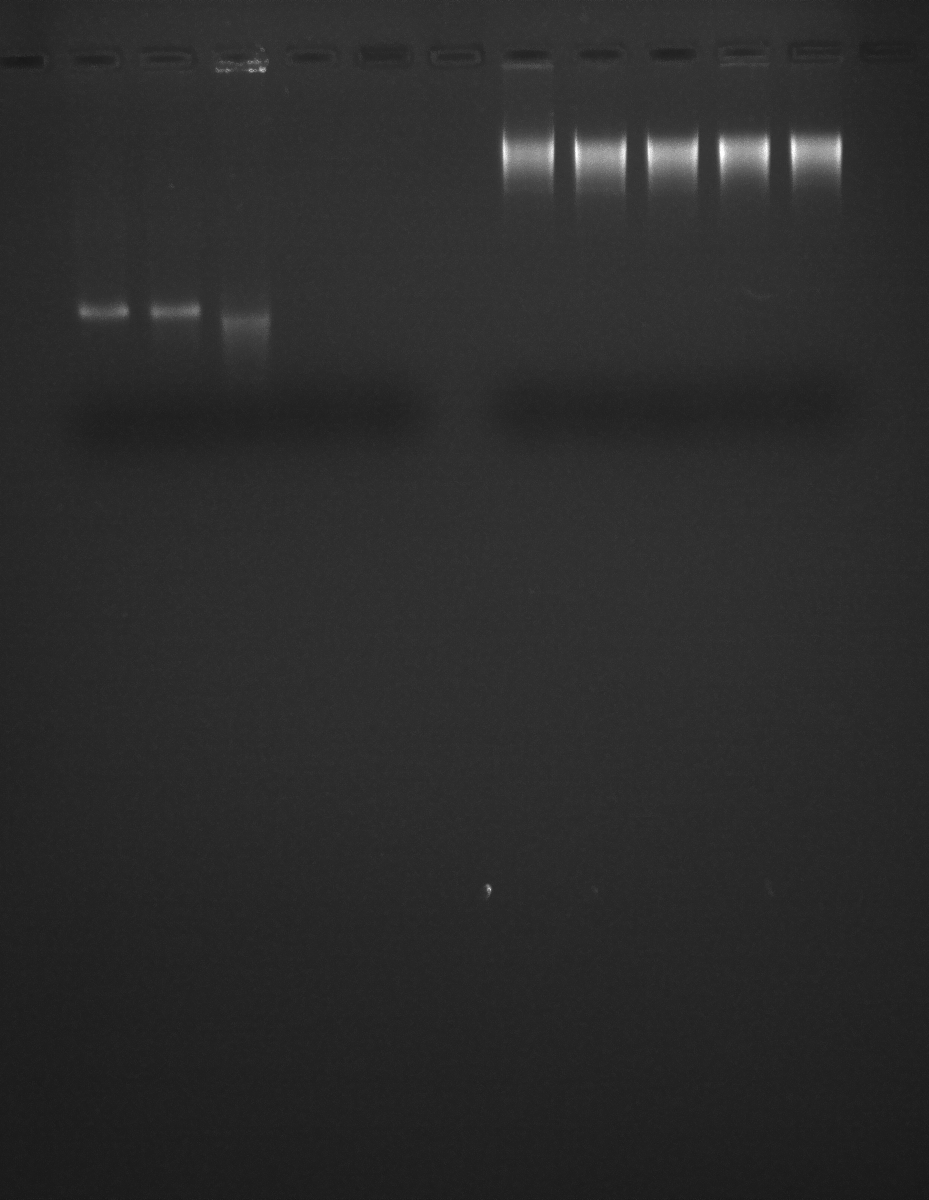

Supplement: Figure 1—figure supplement 1—source data 1. [file elife-111336-fig1-figsupp1-data1.zip › Figure 1-figure supplement 1-source data 1.TIFF]

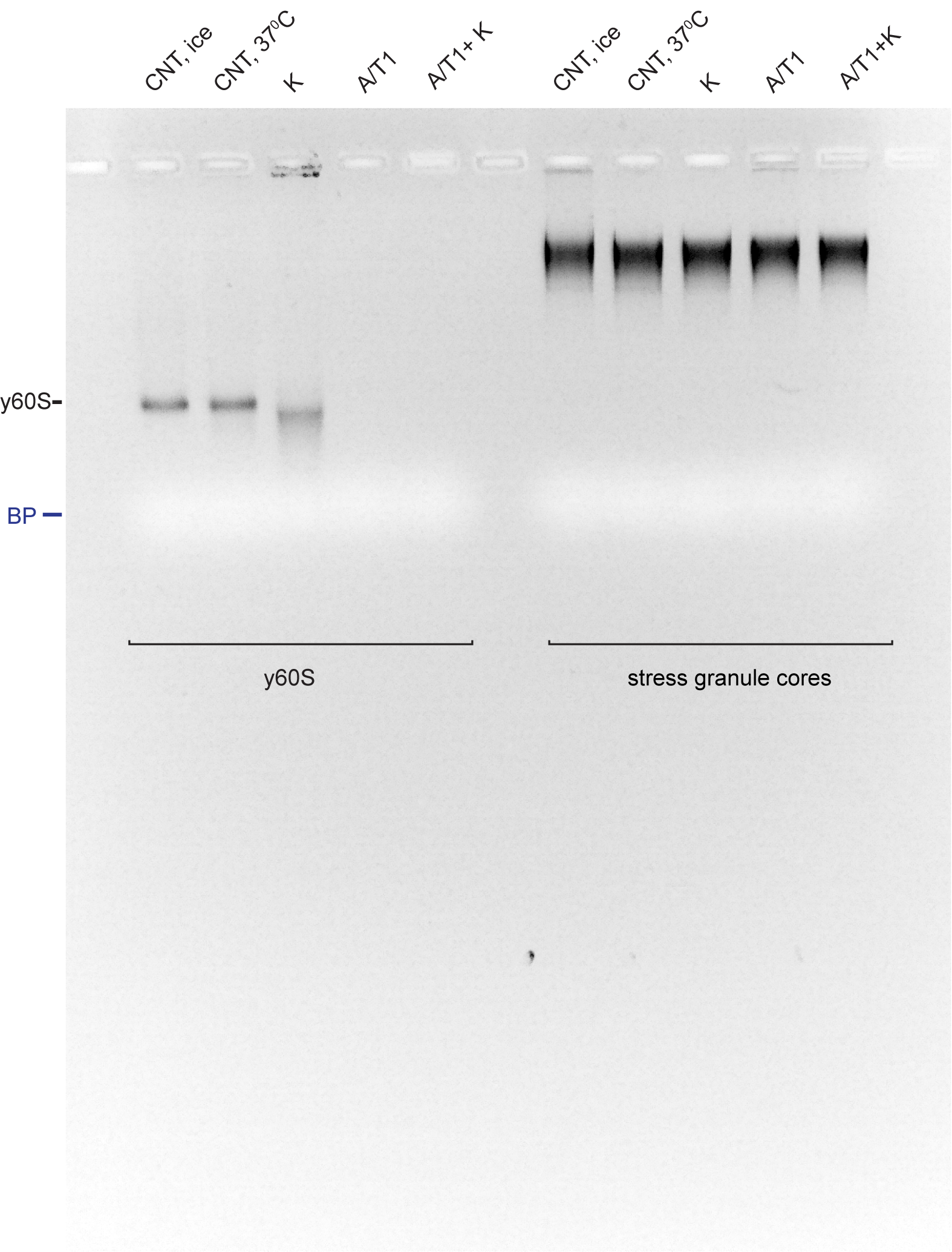

Supplement: Figure 1—figure supplement 1—source data 2. [file elife-111336-fig1-figsupp1-data2.zip › Figure 1-figure supplement 1-source data 1_labeled.tif]

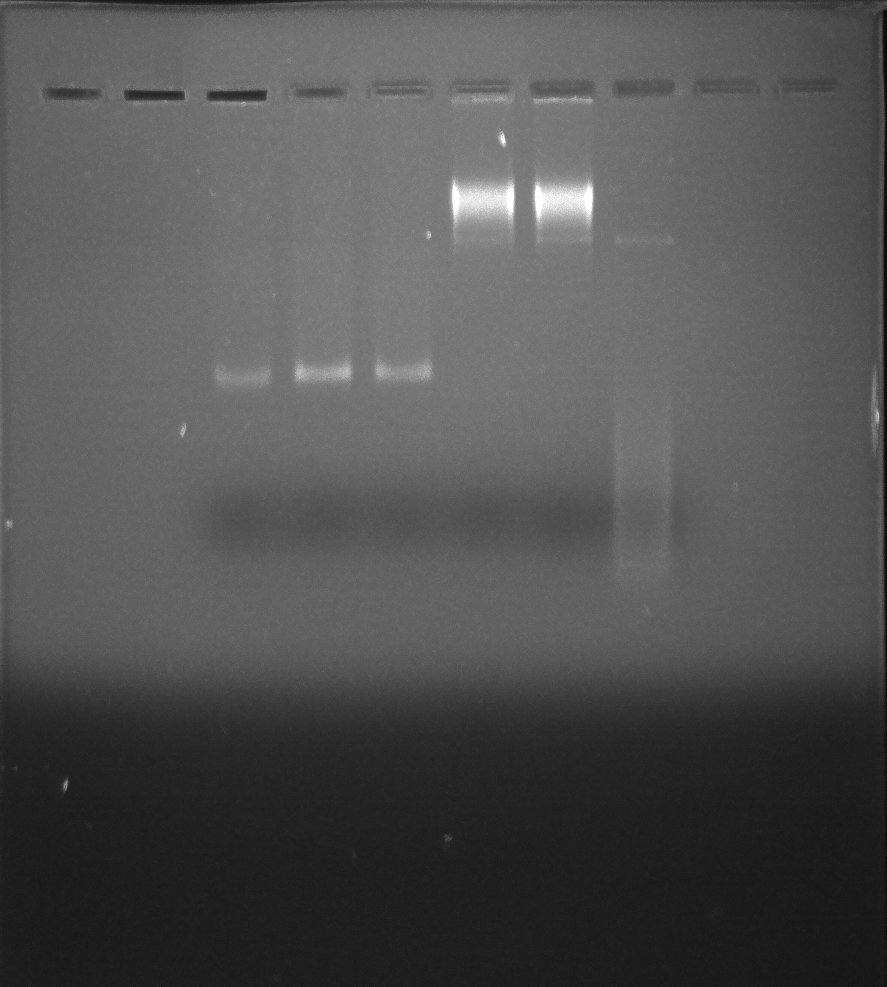

Supplement: Figure 1—figure supplement 1—source data 3. [file elife-111336-fig1-figsupp1-data3.zip › Figure 1-figure supplement 1-source data 2.TIF]

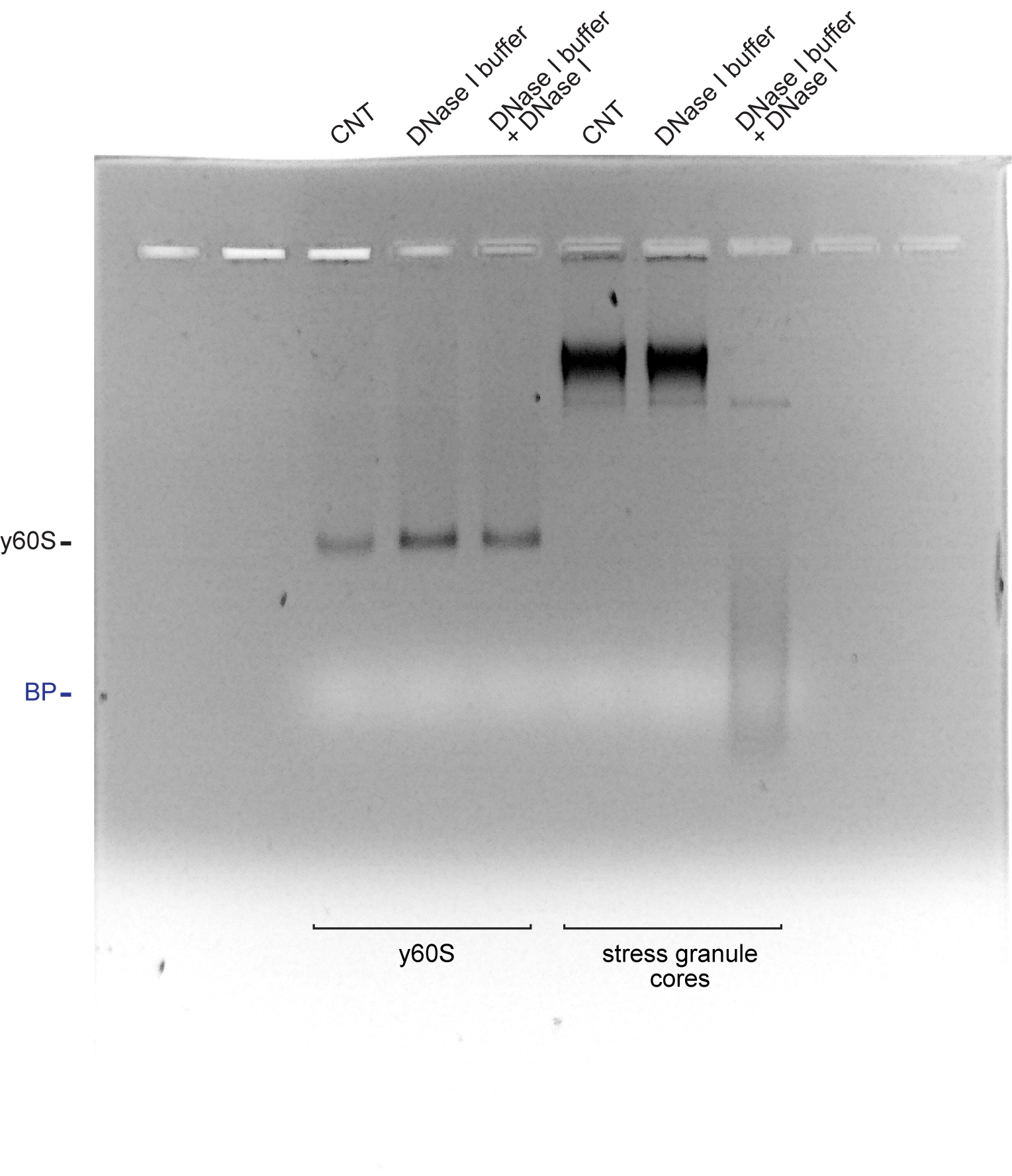

Supplement: Figure 1—figure supplement 1—source data 4. [file elife-111336-fig1-figsupp1-data4.zip › Figure 1-figure supplement 1-source data 2_labeled.tif]

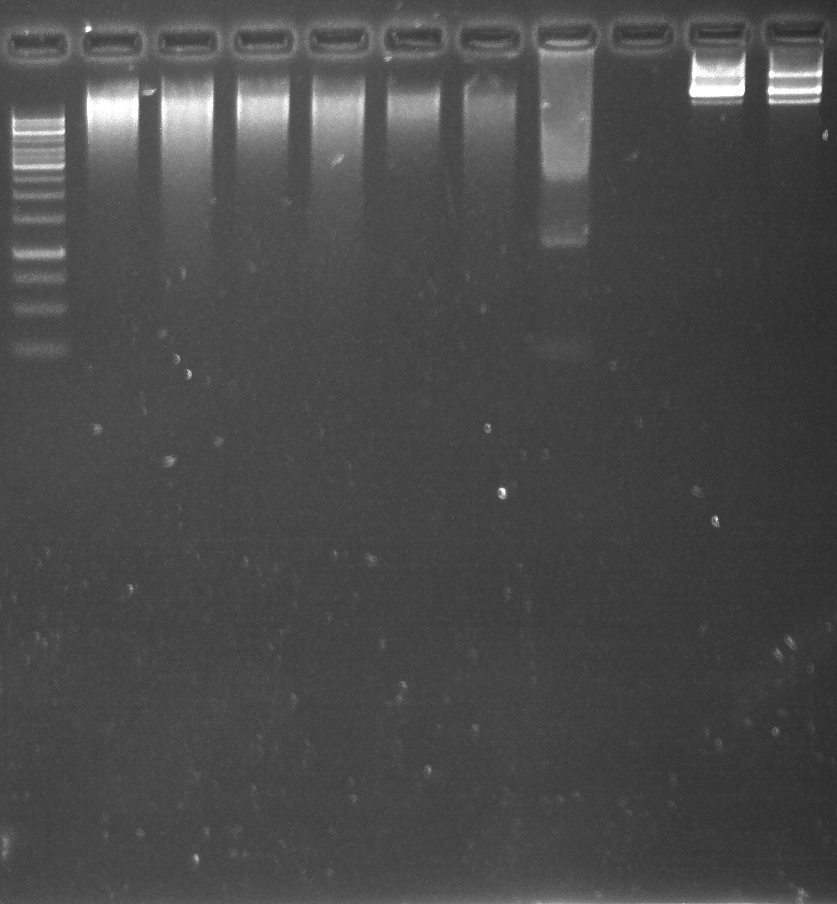

Supplement: Figure 1—figure supplement 1—source data 5. [file elife-111336-fig1-figsupp1-data5.zip › Figure 1-figure supplement 1-source data 3.tif]

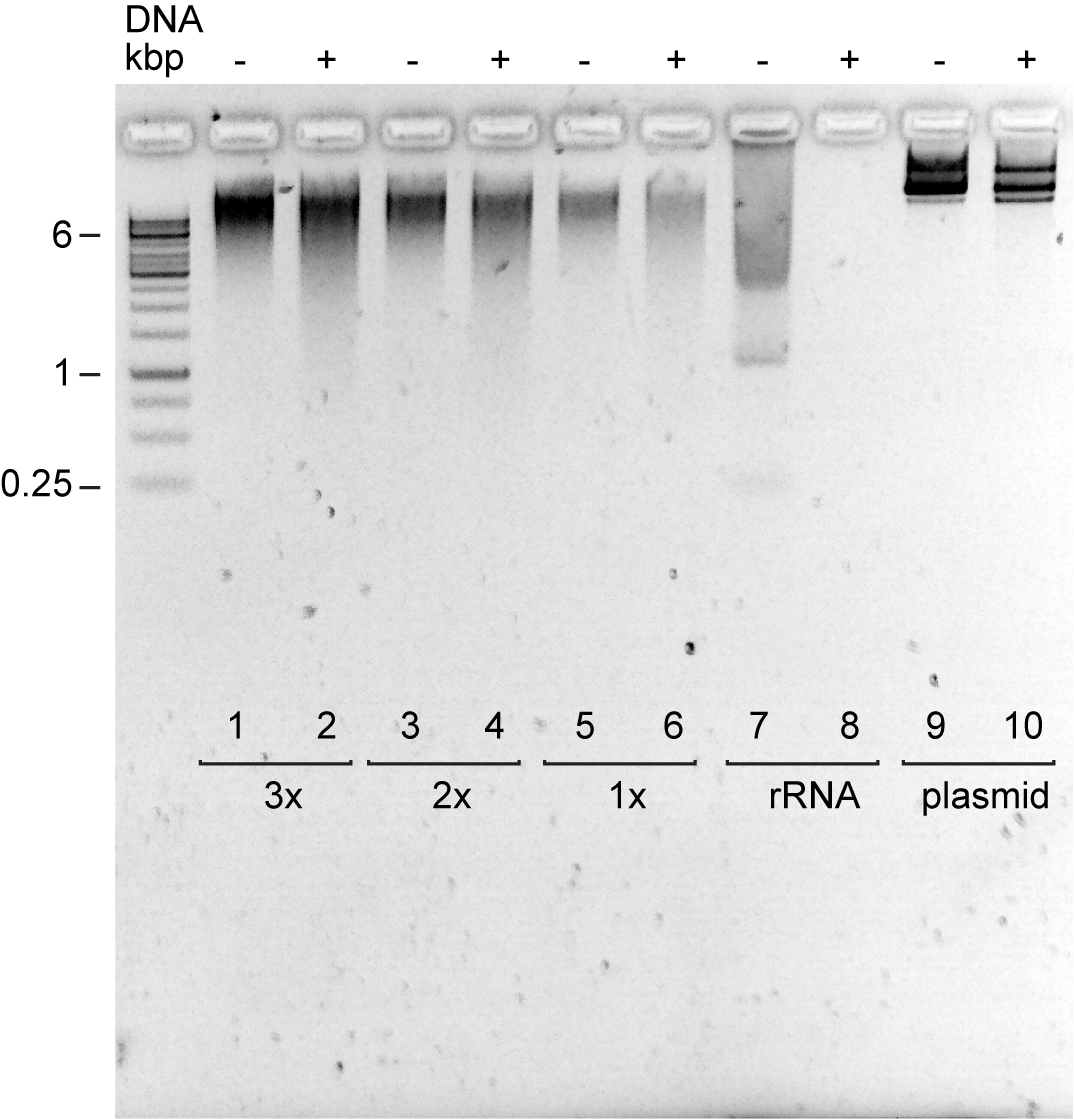

Supplement: Figure 1—figure supplement 1—source data 6. [file elife-111336-fig1-figsupp1-data6.zip › Figure 1-figure supplement 1-source data 3_label.tif]
