# Supplementary material for: Cytoplasmic circular dsDNA is a key constituent of stress granules
Source: eLife. 2026 Jul 13;15:RP111336. doi: 10.7554/eLife.111336 (PMC13363216; doi:10.7554/eLife.111336)
Supplement: Supplementary file 1. [file elife-111336-supp1.docx]

**Supplementary File 1**

**Antibodies used in IF and HCR IF light microscopy imaging of HEK293T cells.**

| **Primary Antibody [dilution]** | **Secondary Antibody [dilution]** | **Amplifier** |
| --- | --- | --- |
| Anti-VCP, mouse, monoclonal [1:100]  (Santa Cruz Biotechnology, Cat# sc-57492) | Anti-mouse, donkey [1:500], B5 initiator  Molecular Instruments, Inc. | B5-647 |
| Anti-histone H4, rabbit, monoclonal  [1:70 – 1:100]  (Abcam, Cat# ab177840) | Anti-rabbit, donkey [1:500], B1 initiator  Molecular Instruments, Inc. | B1-488 |
| Anti-histone H3.1, rabbit, monoclonal [1:30 to 1:90]  (Novus Biologicals, Cat# NBP3-26228) | Anti-rabbit, donkey [1:500], B1 initiator  Molecular Instruments, Inc. | B1-488 |
| Anti-G3BP, mouse, monoclonal [1:200]  (Abcam, Cat# ab56574) | Anti-mouse, donkey [1:500], B5 initiator  Molecular Instruments, Inc. | B5-647 |
| Anti Lamin A + Lamin C, rabbit, monoclonal [1:400]  (Abcam, Cat# ab108595) | Anti-rabbit, donkey [1:800], B1 initiator  Molecular Instruments, Inc. | B1-488 |
| Anti-Caprin1, rabbit, polyclonal [1:400] (Abcam, Cat# ab244360) | Anti-rabbit, donkey [1:750], B4 initiator  Molecular Instruments, Inc. | B4-546 |
| Anti-TOMM20, rabbit, monoclonal [1:250] (Abcam, Cat# ab186735) | Anti-rabbit, donkey [1:500], B1 initiator  Molecular Instruments, Inc. | B1-488 |
| Anti-DNA, mouse, monoclonal [1:200] (Thermo Fisher Scientific, Cat # 690014S) | Anti-mouse, goat [1:800], Alexa Fluor 568  (Invitrogen, Cat# A11004) | N/A |
