# Supplementary material for: Cytoplasmic circular dsDNA is a key constituent of stress granules
Source: eLife. 2026 Jul 13;15:RP111336. doi: 10.7554/eLife.111336 (PMC13363216; doi:10.7554/eLife.111336)
Supplement: Supplementary file 2. [file elife-111336-supp2.docx]

**Supplementary File 2**

**Sequence Read Archive deposition summary for raw DNA sequences.**

| **Bioproject** | **Accession** | **Species** | **Sequencing platform** | **Sample name** |
| --- | --- | --- | --- | --- |
| PRJNA1306188 | SRR34996475 | *S. cerevisiae* | Illumina | Cas9nes_gRNA_01 |
| PRJNA1306188 | SRR34996474 | *S. cerevisiae* | Illumina | Cas9nes_gRNA_02 |
| PRJNA1306188 | SRR34996473 | *S. cerevisiae* | Illumina | dCas9nes_gRNA_01 |
| PRJNA1306188 | SRR34996472 | *S. cerevisiae* | Illumina | dCas9nes_gRNA_02 |
| PRJNA1306188 | SRR34996471 | *S. cerevisiae* | Illumina | No_tranform_01 |
| PRJNA1306188 | SRR34996470 | *S. cerevisiae* | Illumina | No_tranform_02 |
| PRJNA1306188 | SRR34996469 | *S. cerevisiae* | Illumina | Cas9nes_01 |
| PRJNA1306188 | SRR34996468 | *S. cerevisiae* | Illumina | Cas9nes_02 |
| PRJNA1306188 | SRR35009179 | *S. cerevisiae* | Illumina | Cas9nes_gRNA_CHD1nes_01 |
| PRJNA1306188 | SRR35009178 | *S. cerevisiae* | Illumina | Cas9nes_gRNA_CHD1nes_02 |
| PRJNA1306188 | SRR35009463 | *S. cerevisiae* | Illumina | ySCGs_Illumina_01 |
| PRJNA1306188 | SRR35009462 | *S. cerevisiae* | Illumina | ySCGs_Illumina_02 |
| PRJNA1306188 | SRR35009461 | *S. cerevisiae* | ONT | ySGCs_ONT_01 |
| PRJNA1306188 | SRR35009460 | *S. cerevisiae* | ONT | ySGCs_ONT_02 |
| PRJNA1305524 | SRR35178678 | *H. sapiens* | ONT | 7E_Dna_nanopore |
| PRJNA1305524 | SRR35178677 | *H. sapiens* | ONT | 8E_Dna_nanopore |
| PRJNA1305524 | SRR35178676 | *H. sapiens* | ONT | 9E_Dna_nanopore |
| PRJNA1305524 | SRR35178675 | *H. sapiens* | Illumina | 7E_Dna_Illumina |
| PRJNA1305524 | SRR35178674 | *H. sapiens* | Illumina | 8E_Dna_Illumina |
| PRJNA1305524 | SRR35178673 | *H. sapiens* | Illumina | 9E_Dna_Illumina |
| PRJNA1305524 | SRR34981497 | *H. sapiens* | ONT | hSGCs 1_early_ONT |
| PRJNA1305524 | SRR34981496 | *H. sapiens* | ONT | hSGCs 2_late_ONT |
| PRJNA1305524 | SRR34981495 | *H. sapiens* | ONT | hSGCs 3_early_ONT |
| PRJNA1305524 | SRR34981494 | *H. sapiens* | ONT | hSGCs 4_late_ONT |
| PRJNA1305524 | SRR34981493 | *H. sapiens* | ONT | hSGCs 5_early_ONT |
| PRJNA1305524 | SRR34981492 | *H. sapiens* | ONT | hSGCs 6_late_ONT |
| PRJNA1305524 | SRR35328255 | *H. sapiens* | Illumina | hSGCs 1_early_Illumina |
| PRJNA1305524 | SRR35328254 | *H. sapiens* | Illumina | hSGCs 2_late_Illumina |
| PRJNA1305524 | SRR35328253 | *H. sapiens* | Illumina | hSGCs 3_early_Illumina |
| PRJNA1305524 | SRR35328252 | *H. sapiens* | Illumina | hSGCs 4_late_Illumina |
| PRJNA1305524 | SRR35328251 | *H. sapiens* | Illumina | hSGCs 5_early_Illumina |
| PRJNA1305524 | SRR35328250 | *H. sapiens* | Illumina | hSGCs 6_late_Illumina |
