# Supplementary material for: Cytoplasmic circular dsDNA is a key constituent of stress granules
Source: eLife. 2026 Jul 13;15:RP111336. doi: 10.7554/eLife.111336 (PMC13363216; doi:10.7554/eLife.111336)
Supplement: Supplementary file 3. [file elife-111336-supp3.docx]

**Supplementary File 3**

**Cloning materials for CRISPR targeting cytoplasmic eccDNA with Ty1 in yeast.**

| **Plasmid Name** | **Description** |
| --- | --- |
| pML104 | Original (Addgene #67638)  GAP-Cas9_NLS_  Scaffold (tracrRNA) under SNR52  No crRNA |
| pML104-GAL1-Cas9_NES_-Scaffold | GAL1-Cas9_NES_  Scaffold RNA (tracrRNA) / SNR52  No crRNA |
| pML104-GAL1-Cas9_NES_ | GAL1-Cas9-NES  No scaffold RNA  No crRNA |
| pML104-GAL1-Cas9_NES_-Ty1 | GAL1-Cas9-NES-Ty1  crRNA-1: SNR52-Ty11  crRNA-2: SUP4-Ty12^HDV^ (minus MIII insert) |
| pML104-GAL1-Cas9_NES_-Ty1-MIII | GAL1-Cas9-NES-Ty1  crRNA-1: SNR52-Ty11  crRNA-2: SUP4-Ty12^HDV-MIII^ (plus MIII insert) |
| pML104-GAL1-dCas9_NES_-Ty1 | GAL1-dCas9-NES-Ty1  crRNA-1: SNR52-Ty11  crRNA-2: SUP4-Ty12^HDV^ |
| pML104-GAL1-Cas9_NES_-mCherry_NES_-Ty1-MIII | GAL1-Cas9-NES-mCherry-NES-Ty1  crRNA-1: SNR52-Ty11  crRNA-2: SUP4-Ty12^HDV-MIII^ (plus MIII insert) |
