# Supplementary material for: Cytoplasmic circular dsDNA is a key constituent of stress granules
Source: eLife. 2026 Jul 13;15:RP111336. doi: 10.7554/eLife.111336 (PMC13363216; doi:10.7554/eLife.111336)
Supplement: Supplementary file 4. [file elife-111336-supp4.docx]

**Supplementary File 4**

**Primers and fragments designed for modification of the pML104 backbone.**

| **Name** | **Sequence** |
| --- | --- |
| NES-For | 5’- cagatttccgagttttctaaacgcgtcattctcgctgat |
| NES-Rev | 5’- tgccaatctaaacgataccacggccgctctagagaaatgg |
| GAL1-For | 5’- gtatcgataagcttgatatcgaattcagtctactagtcagttcgagtttacg |
| GAL1-Rev | 5’- tttctcgtgataggccacctcgtcgacgatattaccgaagatgg |
| CON-SNR52 | 5’-ggccgtggtatcgtttagattggcaattacagtgtcttagctcacatgcttataactaattacatgactcgaagacataaaaaacaaaaaaagatcatttatctttcactgcggagaagtttcgaacgccgaaacatgcgcaccaactttcacttctacagcgtttgaccaaaatcttttgaacagaacattgtagggtgtgaaaaaatgcgcacctttaccg |
| CON-For | 5’- cgagcaaatgcctgcaaatcgctccccatttctctagagcggccgtggtatcgtttagattgg |
| CON-Rev | 5’- gaagtacaactctagattttgtagtgccctcttgggctagcggtaaaggtgcgcattttttcac |
| MIII(U10A) | 5’-GTACGAAGGAAGGTTTGGTATGGGGTAGTTGTCGTAC-3’ |
| Ty1-2-MIII-HDV | 5’-agacataaaaaacaaaaaaagcaccgactcggtgccactttttcaagttgataacggactagccttattttaacttgctatttctagctctaaaacGGTCTTTATATAGACCAGGAaaa**gtcccattcgccacc**GTACGACAACTACCCCATACCAAACCTTCCTTCGTAC**ggtgttgcccagccggcgccagcgaggaggctgggaccatgccgg**ccatctctcccgggggcgagtcgaacgcccgatctcaagatttcgtagtgataaattacagtcttgcgccttaaaccaacttggctaccgagagtatttaattgttgaagaaagagtatactacataacacatata -3’ |
| Ty12-For | 5’- gcatgaggtcgctcttattgaccacacctctaccggcatgagacataaaaaacaaaaaaagcaccgact |
| Ty11-For | 5’- gatcg**accaggtctttatatagacc**-3’ |
| Ty11-Rev | 5’- **ggtctatataaagacctggt**c-3’ |
| Ty12-Rev | 5’- tgagctaagacactgtaattgccaatctaaacgataccactatatgtgttatgtagtatactctttcttcaacaattaaatactctcg |
| a29c-s | 5'-gtattctatcggactggccatcgggactaatagcg-3' |
| a29c-as | 5'-cgctattagtcccgatggccagtccgatagaatac-3' |
| c2518g-a2519c-s | 5'-ctctgagggacgatggcgtccacgtcgtagtc-3' |
| c2518g-a2519c-as | 5'-gactacgacgtggacgccatcgtccctcagag-3' |
