# Supplementary material for: Cytoplasmic circular dsDNA is a key constituent of stress granules
Source: eLife. 2026 Jul 13;15:RP111336. doi: 10.7554/eLife.111336 (PMC13363216; doi:10.7554/eLife.111336)
Supplement: Supplementary file 5. [file elife-111336-supp5.docx]

**Supplementary File 5**

**Addgene deposit IDs for plasmids used in the study.**

| **Name** | **Sequence ID [deposit]** |
| --- | --- |
| pML104_Gal1_Cas9NES_Ty1MIIIA10U | 215655 [83841] |
| pML104_Gal1Cas9NES_Ty1 | 215656 [83841] |
| pML104_Gal1dCas9NES_Ty1 | 215658 [83841] |
| pML104-Gal1Cas9NES | 215659 [83841] |
| pBY011_Flag_CHD1_NES2 | 215660 [83841] |
| pML104_Ty1_11_12 | 246009 [86470] |
| pML104_Gal1_Cas9NLS | 246012 [86470] |
| pML104_Gal1_Flag_CHD1NES2 | 246015 [86470] |
| pBY011_NES_GCN5_NES_MYC | 246016 [86470] |
