# Supplementary material for: Cytoplasmic circular dsDNA is a key constituent of stress granules
Source: eLife. 2026 Jul 13;15:RP111336. doi: 10.7554/eLife.111336 (PMC13363216; doi:10.7554/eLife.111336)
Supplement: Peer review history

# Editor report: eLife Assessment

**DOI:** 10.7554/eLife.111336.2.sa0

**Authors:** Jungsan Sohn

**Affiliations:** Johns Hopkins University School of Medicine United States

**Keywords:** Convincing, Important

This **important** study demonstrates that extrachromosomal circular DNA and chromatin-associated proteins are components of stress granules. The data from a range of cellular and microscopy approaches are **convincing**, but the main conclusions would be further strengthened by demonstrating functional relevance and by extending the analysis to additional cell types. This paper will be of broad interest to cell biologists and those studying stress granule formation.

# Referee report: Reviewer #1 (Public review)

**DOI:** 10.7554/eLife.111336.2.sa1

Summary:

In this manuscript, Demeshkina and Ferré-D'Amaré showed that extrachromosomal circular DNA (eccDNA) and chromatin-associated proteins are present in stress granules, based on proteomic and sequencing analyses. Using HCR-FISH combined with imaging, the authors showed the colocalization of eccDNA with stress granule proteins. Furthermore, they found that CRISPR machinery targeting the eccDNA component of stress granules disrupts stress granule assembly, and that this effect is largely independent of Cas9 endonuclease activity. Notably, expression of cytoplasmic chromatin factors restores stress granule formation in the presence of CRISPR machinery in yeasts. This also rescues the growth defect caused by hypoxic stress, which correlates with impaired stress granule formation. Together, this manuscript provides insight into the presence of eccDNA in cytoplasmic membraneless organelles, specifically stress granules, and suggests a functional role for eccDNA within these structures under stress conditions.

Strengths:

The authors used a panel of ribonucleases to demonstrate that stress granule cores isolated from yeast and HEK293 cells are resistant to plasmid-safe DNase, an enzyme that does not degrade circular double-stranded DNA. To further support the presence of extrachromosomal circular DNA (eccDNA) in stress granules, they performed Circle-Seq on stress granule cores. The gel electrophoresis and sequencing experiments complement each other well, providing consistent evidence for eccDNA within these granules. Overall, this study provides insight into potential cytoplasmic roles for eccDNA, an area that remains largely unexplored.

Weaknesses:

(1) Figure 1F suggests that stress granule cores are susceptible to DNase I but not to plasmid-safe DNase (psDNase). However, its smearing pattern in the psDNase condition appears similar to that in the DNase I treatment shown in Figure 1E, although psDNase produces more discrete bands. The authors should comment on these differences between Figures 1E and 1F, or consider revising Figure 1F to improve consistency with Figures 1E and 1D.

(2) The authors should clearly define "colocalization". Does it refer to complete spatial overlap between two signals (i.e., VCP and T30), or partial overlap (i.e., AHNAK DNA and G3BP)? Figure 3 and the associated text are descriptive. Quantitative analysis would strengthen the conclusions. For example, the authors could analyze the fraction of molecules localized to stress granules or provide Pearson's correlation coefficient or similar measurements.

(3) The authors used a CRISPR-based approach to target the Ty1 LTR retrotransposon, an abundant stress granule eccDNA, and they observed a loss of stress granule formation. However, this phenotype may be specific to Ty1 eccDNA rather than representative of all eccDNA species present in granules. In particular, the title "Cytoplasmic circular DNA is a key constituent of stress granules" implies a broader role. To support this claim, the authors should consider approaches that more globally deplete eccDNA rather than targeting a single eccDNA.

(4) The authors should provide additional experimental evidence to support the claim that eccDNA is packaged in a chromatin-like state. The rescue of stress granule formation by ectopic expression of modified chromatin-associated proteins (CHD1NES and GCN5NES) following CRISPR treatment does not necessarily demonstrate that eccDNA is packaged like chromatin under basal conditions.

# Referee report: Reviewer #2 (Public review)

**DOI:** 10.7554/eLife.111336.2.sa2

Summary:

The authors report the presence of extrachromosomal circular DNAs (eccDNAs) within the core of stress granules purified from both yeast and mammalian cells.

Strengths:

This study is important for understanding the molecular mechanisms underlying stress granules containing eccDNAs and is likely to have a major impact on future research. A major strength of the study is the extensive experimental validation performed in yeast cells. In particular, cytoplasmic CRISPR-mediated targeting of eccDNAs suppresses stress granule formation and impairs recovery from hypoxic stress in yeast cells.

Weaknesses:

The conclusions would be further strengthened by validating the functional findings in an additional model system, such as mammalian cells.

Comments:

(1) Section: "Stress granule cores contain eccDNA"

a) The presence of eccDNAs would be more convincingly demonstrated using an orthogonal validation approach, such as DNA FISH targeting MYC and Centromere 8 (CEN8) on metaphase spreads from HEK293T cells (as performed in PMID: 34819668).

b) The study would also benefit from assessing the presence of eccDNAs in the extracellular medium. For example, DNA could be extracted from conditioned media and analyzed by PCR using primers spanning eccDNA breakpoint junctions (as performed in PMID: 40074906; PMID: 36123406).

(2) Section: "eccDNA-CRISPR abrogates stress granules"

These findings should be further validated under additional stress conditions, such as drug-induced stress (like methotrexate) or nutrient deprivation in the cell medium.

In addition, the same set of experiments should be performed in HEK293T cells to support the broader relevance of the observations.

# Author comment: Author response

**DOI:** 10.7554/eLife.111336.2.sa3

**Authors:** Natalia A Demeshkina, Adrian R Fèrré-D'Amaré

**Affiliations:** NHLBI / NIH Bethesda, MD United States; NHLBI / NIH Bethesda, MD United States

**Public Reviews:**

**Reviewer #1 (Public review):**

Summary:

In this manuscript, Demeshkina and Ferré-D'Amaré showed that extrachromosomal circular DNA (eccDNA) and chromatin-associated proteins are present in stress granules, based on proteomic and sequencing analyses. Using HCR-FISH combined with imaging, the authors showed the colocalization of eccDNA with stress granule proteins. Furthermore, they found that CRISPR machinery targeting the eccDNA component of stress granules disrupts stress granule assembly, and that this effect is largely independent of Cas9 endonuclease activity. Notably, expression of cytoplasmic chromatin factors restores stress granule formation in the presence of CRISPR machinery in yeasts. This also rescues the growth defect caused by hypoxic stress, which correlates with impaired stress granule formation. Together, this manuscript provides insight into the presence of eccDNA in cytoplasmic membraneless organelles, specifically stress granules, and suggests a functional role for eccDNA within these structures under stress conditions.

Strengths:

The authors used a panel of ribonucleases to demonstrate that stress granule cores isolated from yeast and HEK293 cells are resistant to plasmid-safe DNase, an enzyme that does not degrade circular double-stranded DNA. To further support the presence of extrachromosomal circular DNA (eccDNA) in stress granules, they performed Circle-Seq on stress granule cores. The gel electrophoresis and sequencing experiments complement each other well, providing consistent evidence for eccDNA within these granules. Overall, this study provides insight into potential cytoplasmic roles for eccDNA, an area that remains largely unexplored.

Weaknesses:

(1) Figure 1F suggests that stress granule cores are susceptible to DNase I but not to plasmid-safe DNase (psDNase). However, its smearing pattern in the psDNase condition appears similar to that in the DNase I treatment shown in Figure 1E, although psDNase produces more discrete bands. The authors should comment on these differences between Figures 1E and 1F, or consider revising Figure 1F to improve consistency with Figures 1E and 1D.

We suggest that the appropriate comparisons are between the DNase I and psDNase treatments within each figure panel, and not between panels (e.g., Figures 1E vs. 1F). The electrophoretic gels in the different panels were run for different lengths of time, and therefore the comparison between gels would be spurious. In Figure 1E, electrophoresis after DNase I treatment results in a characteristic smear, while after psDNase treatment yields discrete bands (lanes 2–3 vs. 4–5). Electrophoretic conditions for this figure were optimized to minimize diffusion and allow quantitative evaluation. The electrophoresis shown in Figure 1F, which compares yeast and mammalian stress granule core nucleic acids, was run for a longer period — as evidenced by the greater migration distance from the loading wells — yet still clearly shows the same qualitative difference between DNase I (smear, lane 3) and psDNase (discrete bands, lanes 1–2) treatments for the yeast samples. The apparent discrepancy noted by the referee therefore simply reflects the difference in electrophoretic conditions between the gels shown in the two separate figure panels.

(2) The authors should clearly define "colocalization". Does it refer to complete spatial overlap between two signals (i.e., VCP and T30), or partial overlap (i.e., AHNAK DNA and G3BP)? Figure 3 and the associated text are descriptive. Quantitative analysis would strengthen the conclusions. For example, the authors could analyze the fraction of molecules localized to stress granules or provide Pearson's correlation coefficient or similar measurements.

In our considered opinion, categorizing colocalization as either "partial" or "complete" implies a level of molecular precision that is physically unattainable at the resolution limits of any current light microscopy modality, and would therefore be misleading. Our approach employs super-resolution confocal laser scanning microscopy (Airyscan) with hybridization chain reaction fluorescence in situ hybridization (HCR-FISH) or with immunofluorescence. The detection method used offers higher spatial resolution and signal-to-noise ratio than single-point detector/physical pinhole confocal (or widefield epifluorescence) microscopy used in most prior stress granule studies. Despite these enhancements, the system retains inherent diffraction-imposed limits: a lateral (XY) resolution of ~130 nm and an axial (Z) resolution of ~350–400 nm, defining the minimum separable distance between two fluorescent signals. Structures smaller than these thresholds remain unresolved within a single point spread function (PSF) maximum – a volume sufficiently large to simultaneously accommodate multiple stress granule cores or tens of thousands of individual proteins (such as G3BP) and dozens of nucleic acid molecules several thousand nucleotides in length. Consequently, any detected fluorescence signal may represent the superimposition of a large and indeterminate number of individual molecules or particles. True molecular interaction analysis remains for future studies using technologies with angstrom resolution (e.g., cryo-electron tomography, cryo-EM, X-ray crystallography, smFRET, EPR, NMR, etc.). Metrics such as Pearson's correlation coefficient report solely on the degree of signal overlap at the PSF scale (hundreds of nanometers) and would not provide any insight beyond what is already conveyed by our data.

(3) The authors used a CRISPR-based approach to target the Ty1 LTR retrotransposon, an abundant stress granule eccDNA, and they observed a loss of stress granule formation. However, this phenotype may be specific to Ty1 eccDNA rather than representative of all eccDNA species present in granules. In particular, the title "Cytoplasmic circular DNA is a key constituent of stress granules" implies a broader role. To support this claim, the authors should consider approaches that more globally deplete eccDNA rather than targeting a single eccDNA.

We respectfully disagree with the referee that further depletion of eccDNA would alter our conclusions. A central finding of our study is that stress granules can be abrogated cytoplasmically by co-expressing a Cas9 endonuclease, active or inactivated by point mutations (D10A /H840A), and a gRNA (which is itself a fusion of the crRNA and trcrRNA, natively separate RNAs in the source bacterium). We show in Figure 4 that when the gRNA targets the Ty1 sequences, endonucleolytically active holoenzyme co-expression in the cytoplasm results in loss of the corresponding eccDNAs, as assayed by sequencing of the relevant cytoplasmic fractions. Critically, when a catalytically inactive Cas9 protein (dCas9) is co-expressed with the gRNA instead of the wild-type endonuclease, depletion of the eccDNAs containing Ty1 sequences no longer takes place (Figures 4D and 4E), but stress granule formation is still abrogated (Figure 4C).

In our manuscript, we indicated (as "data not shown”) that co-expression with Cas9 of a gRNA "targeting" a sequence that is absent from the *S. cerevisiae* genome still results in abrogation of stress granule formation. These data are shown in Author response image 1. The gRNA is targeted to the sequence 5’-agaatcgatgcattt, which is absent in the genome of the yeast strain used.

**Author response image 1.** 

It follows from our experiments that stress granule abrogation (1) is not a result of the catalytically active Cas9 endonuclease; (2) is not a result of the presence of a gRNA-directed but catalytically inactive Cas9 holoenzyme, but (3) is the result of the presence of a CRISPR holoenzyme (as defined above) in the cytoplasm.

To reiterate, abrogation of stress granules occurs when a Cas9-gRNA complex is present in the cytoplasm, regardless of whether the nuclease activity exists, or the gRNA targets a sequence that is present in the genome. Importantly, the holoenzyme is required for this phenomenon: presence of the endonuclease or the gRNA alone does not abrogate stress granule formation (Figures S5).

It is because of this unexpected observation that we next hypothesized that activities of the Cas9-gRNA complex other than sequence-specific gRNA-targeted endonucleolytic activity is driving the suppression of stress granule formation. The best documented such activity is DNA sequence sampling (1-dimensional diffusion). We think that 1-dimensional diffusion of the Cas9-gRNA holoenzyme is displacing from the cytoplasmic eccDNA interactors whose association with the DNA is required to drive stress granule assembly. The fact that the stress-granule suppressive effect of cytoplasmic Cas9-gRNA expression can itself be suppressed by two completely unrelated proteins whose only shared feature is action on chromatin (CHD1 and GCN5) strongly supports this hypothesis (Figures 4G, 4H and S6; also response to point 4, below), in addition to confirming that cytoplasmic eccDNA is packaged by histones in a conformation that CHD1 and GCN5 can both recognize.

(4) The authors should provide additional experimental evidence to support the claim that eccDNA is packaged in a chromatin-like state. The rescue of stress granule formation by ectopic expression of modified chromatin-associated proteins (CHD1NES and GCN5NES) following CRISPR treatment does not necessarily demonstrate that eccDNA is packaged like chromatin under basal conditions.

We would like to reiterate the temporal order in our experimental design (detailed in full in Methods and summarized in Results). Cas9_NES_-gRNA and CHD1_NES_ (or GCN5_NES_) were expressed simultaneously (not sequentially) in the cytoplasm. This was intentional, so as to give each player ample opportunity to engage its preferred substrate under non-stress conditions, prior to the brief oxidative stress. The referee appears to believe that cytoplasmic eccDNA was pre-exposed to Cas9_NES_-gRNA, and then the bound endonuclease challenged with chromatin-modifying enzymes.

Our experimental design accounts for the contrasting substrate specificities of CRISPR and chromatin-modifying enzymes. Cas9-gRNA (holoenzyme) binds to nucleosome-free DNA with sub-nanomolar dissociation constant (Kd 0.1–1 nM) but its association with chromatinized DNA is impeded 5- to 100-fold (Isaac et al., 2016; Yarrington et al., 2018; Strohkendl et al., 2021). In contrast, whereas CHD1 binding to DNA is strictly nucleosome-dependent — its chromodomains actively block engagement with protein-free DNA (Hauk et al., 2010), and its productive binding (Kd 10–200 nM) relies on obligate multivalent contacts with the histone octamer, H4 tail, and wrapped DNA (Farnung et al., 2017; Sundaramoorthy et al., 2018).

Our observation that stress granule formation was unperturbed following oxidative stress is most parsimoniously interpreted as CHD1_NES_ outcompeting the CRISPR machinery for cytoplasmic binding to eccDNA by virtue of the latter existing in a histone-bound state that is recognized as chromatin by CHD1 –simultaneously favoring CHD1_NES_ engagement and impeding Cas9 access. Thus, our experiment in effect employs stress granule formation as a readout for differential binding to chromatin or chromatin-like eccDNA.

Farnung, L., Vos, S.M., Wigge, C., and Cramer, P. (2017). Nucleosome-Chd1 structure and implications for chromatin remodelling. Nature, 550(7677), 539–542.

Hauk, G., McKnight, J.N., Nodelman, I.M., and Bharat, T.A.M. (2010). The chromodomains of the Chd1 chromatin remodeler regulate DNA access to the ATPase motor. Mol Cell, 39(5), 711–723.

Isaac, R.S., Jiang, F., Doudna, J.A., Lim, W.A., Narlikar, G.J., and Bhatt, D.L. (2016). Nucleosome breathing and remodeling constrain CRISPR-Cas9 function. Nature Struct Mol Biol, 23(12), 1097–1103.

Strohkendl, I., Saifuddin, F.A., Gibson, B.A., Bhatt, D.L., Russell, R., and Bharat, T.A.M. (2021). Inhibition of CRISPR-Cas9 by bacteriophage-encoded proteins. Mol Cell, 81(8), 1665–1679.

Sundaramoorthy, R., Hughes, A.L., Singh, V., Wiechens, N., Ryan, D.P., El-Mkami, H., Petoukhov, M., Svergun, D.I., Treutlein, B., Sproll, P., and Owen-Hughes, T. (2018). Structural reorganization of the chromatin remodeling enzyme Chd1 upon engagement with nucleosomes. eLife, 7, e35720.

Yarrington, R.M., Verma, S., Schwartz, S., Trautman, J.K., and Carroll, D. (2018). Nucleosomes inhibit target cleavage by CRISPR-Cas9 in vivo.PNAS, 115(38), 9450–9455.

**Reviewer #2 (Public review):**

Summary:

The authors report the presence of extrachromosomal circular DNAs (eccDNAs) within the core of stress granules purified from both yeast and mammalian cells.

Strengths:

This study is important for understanding the molecular mechanisms underlying stress granules containing eccDNAs and is likely to have a major impact on future research. A major strength of the study is the extensive experimental validation performed in yeast cells. In particular, cytoplasmic CRISPR-mediated targeting of eccDNAs suppresses stress granule formation and impairs recovery from hypoxic stress in yeast cells.

Weaknesses:

The conclusions would be further strengthened by validating the functional findings in an additional model system, such as mammalian cells.

Comments:

(1) Section: "Stress granule cores contain eccDNA"

(a) The presence of eccDNAs would be more convincingly demonstrated using an orthogonal validation approach, such as DNA FISH targeting MYC and Centromere 8 (CEN8) on metaphase spreads from HEK293T cells (as performed in PMID: 34819668).

The relationship between eccDNA dynamics and stress granule assembly across distinct cell cycle phases remains an important and poorly explored question. To our knowledge, no published data currently describe how stress response mechanisms are regulated during mitotic division, particularly in metaphase. Our identification of eccDNA as a component of stress granule cores can provide a first tractable framework to investigate this relationship. However, a systematic and in-depth characterization of this phenomenon warrants a dedicated future investigation.

(b) The study would also benefit from assessing the presence of eccDNAs in the extracellular medium. For example, DNA could be extracted from conditioned media and analyzed by PCR using primers spanning eccDNA breakpoint junctions (as performed in PMID: 40074906; PMID: 36123406).

We agree with the referee that eccDNA biology represents a fascinating and rapidly evolving area of research, particularly given the emerging role of eccDNA in oncogenesis. In this context, our identification of eccDNA as a core structural component of stress granules opens a novel avenue for exploring the connection between stress-dependent translational regulation and disease-associated eccDNA dynamics. While we acknowledge the importance of this direction, a rigorous investigation of this relationship requires extensive multifaceted experimentation that falls beyond the scope of the current study.

(2) Section: "eccDNA-CRISPR abrogates stress granules"

These findings should be further validated under additional stress conditions, such as drug-induced stress (like methotrexate) or nutrient deprivation in the cell medium. In addition, the same set of experiments should be performed in HEK293T cells to support the broader relevance of the observations.

We agree with the referee that the composition and dynamics of stress granules arising from different stressors is an important endeavor. However, given the range of stressors documented to result in stress granule formation, those studies fall well beyond the scope of this manuscript. We will note however that the presence of eccDNA in stress granules of yeast and human cells is strong evidence for conservation of function(s). We think that exploration of the role of eccDNA in stress granule formation across the kingdoms of life (stress granules were first observed in heat-shocked tomato plants), cell cycle stages, stressors, etc. will be important research programs for the future.

**Recommendations for the authors:**

**Reviewer #1 (Recommendations for the authors):**

(1) Figures 3D and 3I: The use of magenta and red makes it difficult to distinguish between the two labeled signals. Consider using more contrasting colors to improve visual clarity.

We appreciate the comment regarding color choices in the figures. In our view, magenta and red are sufficiently distinguishable as nucleic acid labels, particularly when combined with the green signal representing G3BP in these panels.

(2) Figures 3F and 3G: Do the authors have an explanation for why AHNAK or MAPT DNA (white) does not colocalize with the anti-DNA immunofluorescence signal?

Immunofluorescence (IF) is standard for detecting protein antigens but has limitations when the target is a non-protein molecule such as DNA, owing to its compacted chromatinized state. Anti-DNA antibodies can miss a significant fraction of their targets because the DNA backbone remains largely inaccessible, a limitation that DNA-FISH overcomes by directly hybridizing probes to denatured DNA sequences with high specificity. The fixation step required for both IF and FISH imaging can introduce additional steric barriers that disproportionately restrict antibody access compared to small nucleic acid probes. Even under optimized conditions, the IF signal with anti-DNA antibodies is inherently reflective of a subset of the total cellular DNA content.

(3) Adding a subtitle on page 12 ("The abundant histones in purified stress granule...") would improve the overall structure and readability of the manuscript.

We think that an additional subtitle would not substantially improve the readability of what is, admittedly, a very dense manuscript that employs a diversity of experimental approaches.

(4) It would strengthen the analysis if statistical significance were included for the different time points in Figure 5C.

We appreciate the reviewer’s suggestion. Figure 5C shows the largest difference at 40–45 hours after stress recovery, which is statistically significant between Cas9NES-gRNA (or dCas9NES-gRNA) and Cas9NES or gRNA only (two-tailed Student’s t-test, *, p ≤ 0.05). All primary experimental data are publicly available (FigShare) so further analyses can be performed by interested future parties.
